# Drainage systems, an occluded source of sanitation related outbreaks

**DOI:** 10.1186/s13690-014-0056-6

**Published:** 2015-02-26

**Authors:** Kristina Blom

**Affiliations:** Medibiome AB, 431 53 Mölndal, Sweden

**Keywords:** Sanitation, Drains, Outbreaks, Infections, Resistance, Pathogens

## Abstract

**Background:**

Drainage systems and its role in sanitation related outbreaks are evident but still occluded once it has been installed. This current review evaluates if drainage systems can cause infections and thus be of clinical concern.

**Method:**

A review of the literature was analyzed. Papers, guidelines, and quality management systems have been considered.

**Results:**

Adequate sanitation is fundamental and a prerequisite for safe life and productivity. In contrast, malfunctioning sanitation has been reported to cause outbreaks all over the world. In areas with no sanitation, diarrheal mortality is high and has been shown to decrease by 36% after interventions to improve sanitation. Often, infections are faeces associated and when present in wastewater and sewage sludge poses a high risk of infection upon exposure. Hence, there are working safety guidelines and in industries where infection reduction is essential strict quality assurance systems, i.e. HACCP (hazard analysis critical control points) and GMP (Good Manufacturing Practice) must be complied. Healthcare has recently taken interest in the HACCP system in their efforts to reduce healthcare associated infections as a response to increasing number of ineffective antibiotics and the threat of mortality rate like the pre-antibiotic era. The last few years have called for immediate action to contain the emergence of increasing resistant microorganisms. Resistance is obtained as a result of overuse and misuse of antibiotics in both healthcare and agriculture. Also, by the discharge of antibiotics from manufacturers, healthcare and society. One mechanism of development of novel resistant pathogens has been shown to be by effortless sharing of genetic mobile elements coding for resistance from microbes in the environment to human microbes. These pathogens have been sampled from the drainage systems. These were noticed owing to their possession of an unusual antibiotic resistance profile linking them to the outbreak. Often the cause of sanitation related outbreaks is due to inadequate sanitation and maintenance. However, in general these infections probably go unnoticed.

**Conclusion:**

Drainage systems and its maintenance, if neglected, could pose a threat in both community and healthcare causing infections as well as emergence of multi-resistant bacteria that could cause unpredictable clinical manifestations.

## Background

Sanitation is defined by the World Health Organization (WHO) as: “*Sanitation generally refers to the provision of facilities and services for the safe disposal of human urine and faeces. Inadequate sanitation is a major cause of disease world-wide and improving sanitation is known to have a significant beneficial impact on health both in households and across communities. The word ‘sanitation’ also refers to the maintenance of hygienic conditions, through services such as garbage collection and wastewater disposal*” (http://www.who.int/topics/sanitation/en/).

Sanitation is in general inadequate in rural areas and in developing countries [1,2] while regarded as safe in the developed countries in the community as well as in healthcare. However, it is not sufficient to have access to water and modern drainage systems unless adequate sanitation is maintained. Quality assurance maintenance work is implemented in pharmaceutical and food industry in order to reduce the risk of exposure to the hazards (e.g. pathogens causing clinical manifestations) in the disposals. However, in the community or in healthcare, sanitation is not prioritized and often forgotten [3], despite that wastewater disposal contains increased level of human microbes and that there are several reports implicating drains as a source of infection (Table 1). For instance, in the community the severe acute respiratory syndrome (SARS) outbreak in 2003 at Amoy Garden was reported by WHO to likely have been caused by faulty plumbing due to lack of maintenance causing dry out traps [4] and in healthcare, Starlander and Melhus reported in 2012 of a minor outbreak of extended spectrum β-lactamase (ESBL) producing *Klebsiella pneumoniae* due to contaminated sink drains [5]. Indeed, drains are implicated as being a source of microbes causing infection both in community and healthcare [6,7]. Breathnach *et al.* further pointed out that proper design and maintenance of the wastewater system are essential to prevent infections at intensive care units (ICU) [7]. However, its impact on infection rates has not yet been fully elucidated.

**Table 1:** **Reports implicating drains as the source of outbreaks**

**Microorganism**	**Reservoir**	**Location**	**References**
SARS	Dry U-traps	Community, Amoy Garden, Hong Kong	WHO [8]
Multidrug resistant *Pseudomonas aeruginosa*	Faulty drains	Hospital-wide and medical unit, England	Breathnach *et al*. [7]
Carbapenem resistant Enterobacteriaceae	Sink drains	ICU, Melbourne, Australia	Kotsanas *et al.* [9]
Several different fungi	Drains in bathrooms Drains in kitchen sinks	Community, Osaka, Japan	Hamada *et al.* [10]
*Pseudomonas aeruginosa*	Sink drains Whirlpool drains	ICU at Burn hospital, Cincinnati, OH	Edmonds *et al*. [11]
*Pseudomonas aeruginosa*	Sink drains	Medical-Surgical ICU, Chicago, IL	Levin *et al.* [12]
*Pseudomonas aeruginosa*	Toilets	Out patients, Tübingen, Germany	Döring *et al.* [13]
*Fusarium* spp.	Plumbing drains	131 buildings from 8 states, US	Short *et al.* [6]
extended-spectrum beta-lactamase-producing (ESBL) Enterobacteriaceae	Sink drains	Cardiac-Surgical ICU, France	Kac *et al*. [14]
*Pseudomonas aeruginosa*	Sink drains	Hematology unit, Lund, Sweden	Dagens Nyheter [15]
ESBL *Klebsiella pneumoniae*	Sink drains	Neurosurgical ICU, Uppsala, Sweden	Starlander *et al*. [5]
*Klebsiella pneumonia* Carbapenem resistant	Sink drains	ICU, Sørlandet, Norway	Tofteland *et al.* [16]
*Pseudomonas aeruginosa*	Drains	ICU, Edinburgh, UK	Gillespie *et al.* [17]
ESBL Enterobacteriaceae	Sink drains	ICU, Tours, France	Roux *et al.* [18]
Carbapenem-resistant *P. aeruginosa*	Unsealed drain	Urology ward, Barcelona, Spain	Peña *et al.* [19]
*P. aeruginosa* with unusual antibiogram	Drains	Neurosurgery ICU, Clichy, France	Bert *et al.* [20]
ESBL *Klebsiella oxytoca*	Sink drains	Hospital, Toronto, Canada	Lowe *et al.* [21]
Foot Mouth Disease virus	Leaking drains	Community, Pirbright, UK	HSE [22]
*P. aeruginosa*	Grooved drainage design	Haemotology, Singapore	Ling and How [23]
Carbapenem resistant *Klebsiella oxytoca*	Drainpipes, traps	ICU, Spain	Vergeres -Lopez *et al.* [24]

In the current paper, different aspects supporting the role of drainage systems and sanitation to cause infections and emergence of resistance are delineated. Aspects considered are from past and recent outbreaks, from developing and developed countries, and also, the ways to prevent spread of bacteria in the working environment, at wastewater treatment plants, and food industry. Presented data will hopefully contribute to a better understanding of the importance of proper sanitation and its maintenance to prevent infections.

## Methods

Papers were found in PubMed and Google Scholar starting 1907 using key words such as sanitation, infection, transmission, drains, sinks, outbreaks, drainage systems, sewage, wastewater treatment plants, bacteria, virus, resistance, antibiotics, biofilm, infection prevention, and health care associated infections. The fact that contamination on surfaces, transmission of pathogens, and infections must be prevented not only in health care but also in industry and in the community led to that experiences from these disciplines were explored if useful also to health care. Internet was searched to find news from community cases and guidelines on what conditions and requirements that e.g. food industry must comply with as well as workers at risk of exposure to sewage. Guidance documents were found at the databases of e.g. World Health Organization, Health and Safety Executive (UK), Centers for Disease Control and Prevention (CDC), Atlanta, GA and quality management systems were found by searching God Manufacturing Practice and hazard analysis critical control points (HACCP). Also, regulations from authorities such as Environmental Protection Agency were considered to be relevant to assess the risk to be exposed to sewage. The search outcome, 71 references, is summarized in this review and describes aspects that support the finding that drainage systems if not properly installed or designed and maintained shall be considered as hazardous that may cause infections and emergence of pathogens armed with additional resistance and virulence as well as other diseases e.g. allergies against mold.

## Results and discussion

### Wastewater as a source of infection

#### Developing countries

In particular developing countries and to some extent also developed countries, rural areas predominantly, sanitation is inadequate resulting in diseases such as infections [2]. It is estimated that there are over 4 billion diarrheal cases per year resulting in 2.2 million deaths [2]. The major cause is exposure to excreta and wastewater where *Vibrio cholerae* and *Salmonella typhi* are notable causes of diarrheal diseases [25,26]. Interventions focusing on sanitation has shown to reduce the percentage in diarrheal morbidity with 36% [27]. Other benefits with improved sanitation are economic and environmental gains [28]. In numbers, the return of investment is huge. Every US$1 invested on sanitation brings a $5.50 return by keeping people healthy and productive. In comparison, poor sanitation, costs countries between 0.5 and 7.2% of their gross domestic product (GDP) [28]. The potential savings and need for better quality in life has resulted in e.g. the UN-Water wastewater task force involving different stakeholders working to improve sanitation [29].

#### Working environment at wastewater treatment plants

In excreta disposal there is a high load of microorganisms such as viruses, bacteria, fungi, and protozoa that could cause infections [30,31]. Based on the study of Korzeniewska and Harnisz, the load of enteric bacteria is probably underestimated due to insufficient detection methods [32]. Furthermore, it has been shown that both air and water are contaminated by bacteria in the sewage at Wastewater Treatment Plants (WWTP) jeopardizing the health of both plant workers and surrounding population [32]. Clearly human excreta must be managed in a safe way to minimize the risk of exposure to microbes of clinical significance. This condition is further realized in regulations and guidance documents by e.g. the Centers for Disease Control and Prevention (CDC), Health Safety Executive (HSE) in UK, and Environmental Protection Agencies (EPA) to assure safe working environment for workers exposed to excreta disposal [33-35]. Sadly, despite abolition of manual scavenging in India, about 1.2 million scavengers work as sanitary workers and face numerous health hazards among them infections [36].

#### Food Industry

In food industry, food safety is essential and regulated. Regulations have been spawn from risk impact assessments (RIA) and cost-benefit analysis [37]. The production is run applying two different systems, hazard analysis critical control points (HACCP) and Good Manufacturing Practice (GMP) intended to assure the safety of food. A major focus is on the reduction of microbes. Noteworthy, is the architectural demands of GMP. GMP focus on e.g. that the facilities must be built and the work must be done so that the risk of contamination is minimized and the quality of the product can be assured. In practice, sinks must be placed strategically to ease the drainage of water during cleaning, and in clean rooms or sterile rooms with the highest clean air requirements, sinks or floor drains are not allowed. Pipes must be made of certain materials and their dimensions must differ from those drainage design requirements in ordinary buildings. Floor drains should be kept to a minimum and must be free from debris, giving off no offensive odors. In addition, all building drains must be cleaned and sanitized on a regular basis. Their design must prevent the possibility of backflow. Open channels should be easy to clean and disinfect. These drains must always be filled with water as a physical barrier and their covers must remain intact. Maintenance must be recorded and continuously monitored. Furthermore, it is essential and mandatory to continuously monitor and include environmental sampling. In the food industry, it has been suggested that if one organism is found in the preparation environment, then there is a 70% chance of it getting into the food (Chris Griffith at IAFP Rome 2007). If this principle holds, then the risk of cross-contamination from the building’s drainage systems to other surfaces and/or individuals at hospitals is indeed high. However, GMP systems are not applied in healthcare. Although, HACCP has been evaluated for its applicability to reduce infections but only a handful studies have been found [38]. Thus, practices or maintenance programs considering the sanitation are pretty much up to each hospital.

### Wastewater as a source of emerging novel pathogens

Sewage sludge can end up on landfills but this is problematic since the leakages from the landfill may reach the groundwater [39]. WWTP then offer better management of the sewage since it has to go through different stabilization treatments in order to produce safe sewage sludge [40]. However, WWTPs are focusing mainly on stabilizing organic residues and heavy metals while microbes are neglected. Sewage sludge commonly contains high load of microbes that are able to infect both humans and animals but currently ways of accurate detection and treatment are lacking [32,41]. Detection with conventional plate count technique is most likely not working since bacteria can exist in viable but non-culturable stage and hence the load will be underestimated [42]. These reservoirs of microbes could then go undetected and be spread to the environment and infect crops, animals, and humans [43]. Yet another spread from the environment to the microbiome can be genetic information coding for resistance and virulence [44-46]. This mode of genetic transfer is most likely to have occurred in the case of the rise of the German isolate of *Escherichia coli* (*E. coli*) that was found to have two different mechanisms of virulence [47]. This isolate belonged to enteroaggregative *E. coli* (EAEC) carrying virulence factors on a plasmid (a mobile genetic material) and it obtained the shiga toxin gene carried on a phage (a mobile genetic element) from a shiga toxin producing *E. coli* (STEC). It caused unpredictable novel clinical manifestations in a severe outbreak in Germany 2011 with nearly 7,000 reported cases and 18 deaths due to gastroenteritis and 35 deaths due to hemolytic uremic syndrome [47]. The ease by that bacteria can share genetic information on mobile elements and the knowledge that genes coding for resistance is coded by mobile genetic elements probably will present further unwanted surprises [48]. The fear of creation of multi-resistant bacteria is enforced by the fact that antibiotics are discharged from manufacturers and hospitals and will stress the bacteria in the sewage or environment to develop and disseminate resistance [46,49,50]. Evidence was presented at the 51st Interscience Conference on Antimicrobial Agents and Chemotherapy that hospital sewage is breeding ground for genetic exchange of resistance between bacteria in the environment and clinical isolates [51]. Furthermore, it was recently proven that resistance cassettes against five classes of antibiotics (β-lactams, aminoglycosides, amphenicols, sulfonamides, and tetracyclines) had a perfect genetic match in a clinical relevant bacteria and an environmental bacteria [52]. This study explained the mechanism of lateral spread but also how antibiotic resistance disseminate and can have clinical implications. In the era of increased antibiotic resistance and less available effective therapies, it is increasingly difficult to treat infections. Still with effective antibiotics, infections remain a primary cause of morbidity and mortality in the developed world [53]. Predictions say that unless immediate and consorted actions are taken we will face mortality rate like the pre-antibiotic era. In relation to this dark prediction, the WHO has stated that: “Antibiotic resistance is one of the greatest threats to global health security extending far beyond the human health sector”. The future looks dark also in respect to the possibility of finding the substance that will kill and not provoke resistance [54]. Therefore the return of investment to develop novel antibiotics is judged not worth the effort, hence few drugs are in the pipeline [54,55]. Instead, focus has been directed to preventive measurements. Infection prevention programs have been enforced by the World Health Organization (WHO), European Commission and the US, and national governments [56-59].

### Drains as source of infection

The clinical impact of drains as reservoirs of microorganisms has not yet been fully explored although it is widely established that human excretions such as faeces, urine, oral-nasal aerosols, and skin flakes will carry microbial burden consisting of bacteria and/or virus. For instance, there are 120 different viruses in human faeces [43]. It is also reported that patient flora can be detected in sinks and building drains [10,12,60]. In a new long-stay hospital, it was discovered that identical strains were found in the sinks as well as in the admitted patients [60]. The major correlating strains were pathogens such as the *Escherichia coli, Klebsiella*, *Pseudomonas* and *Acinetobacter* species, all being gram-negative bacteria, with higher correlation to strains isolated from the throats and intestines of patients. Actually, the major reservoir of multi-drug resistant Gram-negative bacilli is the gut of man and animals. At hospitals, discharge of antibiotics are also high [49]. Clearly, drains are reservoirs for microbes and antibiotic residues. It is also clear that microbes in drains and pipes adheres to the surfaces of drains and draining pipes as microbial biofilms, creating a complex ecosystem of different microbes that are fed by organic and inorganic matters [61]. Hota *et al*. elegantly showed the presence of *Pseudomonas aeruginosa* (*P. aeruginosa*) biofilm in drainage systems and their role in the propagation of an outbreak [61]. It is also known that bacteria such as *S. aureus* promote the transfer of antibiotic resistance to other bacteria when present in biofilm [62]. Certainly, drains seem to act as cradles to the emergence of bacteria armed with abilities to resist multiple antibiotics. The development of resistance is probably enhanced at hospitals due to that more bacteria and more antibiotics are flushed down the drains due to the very nature of hospitals constantly caring for numerous different patients that are ill and treated with antibiotics. Thus, biofilm in building drains, not properly maintained, have the potential of spreading even more resistant bacteria. This was indicated in the extended non-frequent outbreak of Carbapenem resistant *Klebsiella pneumonia* (KPC) at an ICU [16]. The source of transmission was found to be drains that was detected by molecular profiling to be the only source to harbor KPC, persistently. Outbreak with *P. aeruginosa*, revealed that patients were not colonized on admittance, but acquired a multi-resistant *P. aeruginosa* during hospitalization [17]. By running antibiogram and molecular profiling, drains were found to be the only source. Therefore these reservoirs are crucial to control. Maintenance of proper sanitation must then be guarded where seepage and backflow never should occur. However, it seems that the management of maintenance is more difficult in practice than in theory. The difficulty of eradicating antibiotic resistant bacteria from sink drains at an intensive care unit (ICU) was recently reported [9]. Several different cleaning methods were tried including hypochlorite, mechanical, and pressurized steam at a temperature of 170°C. However, none of these methods worked. This highlights the need for physical barriers such as water seals for drains that prevent the exposure to microbes in the drains and a way to control the integrity of the barrier.

### The impact of malfunctioning plumbing

The malfunctioning plumbing was recognized as a problem already during the cholera outbreak in London by J. Snow in the 19^th^ century [63]. Later in 1907, it was shown in a sham study that malfunctioning plumbing created aerosols with microorganisms that could be transmitted to humans both indirectly through environmental surfaces and directly through aerosols and cause disease [64]. Sham studies, also in present time support earlier findings and stress the importance of a physical barrier by a water seal between the drainage systems and surroundings [65]. The creation of aerosols has been shown to occur when the plumbing is not correctly designed or if there are leakages, stops or by dried U-traps. Also, aerosols can be created upon flushing the toilet or in situations when water is poured from taps to sinks and drains [66,67]. As shown in the SARS outbreak at Amoy Gardens, aerosols can enter the ventilation system and be spread to all the other connected rooms igniting a fearsome spread [67]. The outbreak at Amoy Gardens highlights the need for good maintenance practices and water safety programs with recommended actions [68]. In the event of backflow or flooding from the drainage systems of wastewater into the building, prompt actions must be taken to protect health and property [31]. Among public health professionals, it is well known that if wastewater leaks to structures and furnishing harmful substances such as gases and pathogenic microorganisms can enter as well as increased humidity that can promote environmental microorganisms to multiply and cause diseases and mold-associated allergies [31]. Therefore, it is essential that the properties are restored to a dry state as quickly as possible [31]. In recommended guidelines by EPA in the US, these highlight the increased safety risks due to the high threat of infection, in the event of sewage backflow into buildings [31]. Consequences of malfunctioning plumbing can indeed cause outbreaks [7]. In one outbreak in London, 85 patients became infected during 2005 and 2011, with overall mortality at 40%, but for patients with sepsis, mortality was 78%. Yet another outbreak occurred in Southern England with four cases and no deaths. Both outbreaks were caused by multidrug-resistant *P. aeruginosa*. There had been 391 notifications of blockages in the wastewater system at the hospitals each year. Blockages had been due to patient wipes and paper towels causing backflow to toilets and showers, leakages, etc. near clinical areas [7]. It was first after re-plumbing, replacing toilet bowls, and etc., the infection rates were notably reduced. The authors stressed the importance of hospital design and engineering in controlling and preventing infection, a factor that highlights the need for engagement from the clinical staff, engineers, and janitors. Also, to consider, these outbreaks were identified due to the unusual antibiogram of organisms and could thus be linked to the hospital wastewater systems. Clearly, hospital wastewater system could be a source of many cases of infection with different bacteria. Breathnach *et al.* concluded: “However, unless the organisms are distinctive in some way, such as being multiply resistant, or several cases with the same species linked in time or place, it is likely that the source of many such infections will remain unrecognized” [7].

### Maintenance of sanitation is essential

Facts point at that sanitation is essential to maintain, to reduce the risk of infection [1]. In a press release from WHO it stated that in *“absence of proper maintenance and without consistent monitoring, reviewing, enforcing and updating of building standards and practices, inadequate plumbing and sewage systems could continue to enhance the potential of SARS and some other diseases to spread* [4]*”*. Numerous cases from both developing and developed countries describe outbreaks caused by faulty sanitation [2] (Table 1). The problem might be that once sanitation facilities are installed the general opinion seems to regard it as a self-sustaining system that is safe. Furthermore, it is easily forgotten since the drainage systems is out of sight. Thus drainage systems are neglected as potential reservoirs of transmission of microorganisms. It is when they cease to function adequately because of e.g. leakages or blockages that the threats to health becomes a reality and inspections followed by reparations or even total exchange of drainage systems are required [31,69]. The awareness about the impact of inadequate sanitation seems poor. The illnesses are many and affect the health and economy of individuals, society, and nations [31].

### Research need

Interdisciplinary work is needed in order to establish guidelines for healthy sanitation in buildings focusing on the clinical need for infection control supported by architectural design and maintenance practices. To accomplish the task, three aspects important for infection reduction must be evaluated. 1) Detection of pathogen containing aerosols and surface contaminants should be done and further analyzed by molecular profiling and antibiograms in order to trace outbreak related pathogens. 2) Current architectural designs should be evaluated if constructed from an infection reduction perspective. 3) Also, the different maintenance practices should be evaluated and explored how to optimize. Possibly, the GMP guideline in food industry and their demands from an infection reduction perspective could be a good start to learn from. Also, the building and facility sectors should be involved to develop what actions are required to maintain a safe sanitation system and with what detection methods maintenance can be surveyed. One detection method that could meet the demands for accurate monitoring of the status in the drainage systems is a technique based on sonar technology, developed by Gormley *et al*. [70]. The need for guidance is especially urgent within healthcare owing to that at hospitals the majority are patients that are sick with less fit immune system and therefore more receptive to infections and thus in need of antibiotics that are effective.

Further research is needed to explore how aerosols produced with or without faulty drainage systems can be disseminated by existing ventilation system and cause disease. In the case with the SARS outbreak, it was clearly shown that the spread of the virus containing aerosols created from the drains was facilitated by the ventilation system. It was further proven in a sham study that aerosols were created and spread by the airflow upon flushing a toilet [67]. Spread of pathogens from wastewater via the drainage systems and out where no physical barrier exist has also been proven in yet other sham studies [71] evoking further issues such as what are the consequences of letting out gases and aerosols through the roof, possibly next to a ventilation inlet, or if there is a leakage along the drainage systems within the building [71]? Leakage or seepage shall be considered and its consequences analyzed. One possible effect could be that humidity is increased thus promoting microbial growth of for e.g. fungi risking mold in the building and allergies to the inhabitants. The impact of microscopic sized leakage is also of interest to study if these would allow microorganisms being microscopically small; virus being 0.1 μm, bacteria around 1 to 10 μm, and fungi around 10 μm big, to leak. Results may show that it is essential that drainage systems must be completely tight and intact where openings have physical barriers.

## Conclusion

Despite good sanitation, problems arise when the sanitation system is not working properly in case of e.g. leakage and backflow caused by blockages or dried out traps. It is beyond proof that wastewater, sewages, or drains act as cradles for emerging new microorganisms with increasing ability to resist antibiotics and possibly armed with virulence factors obtained from other encountered species [47,50,51]. The cradles consist of microbial biofilm, where transfer of genes coding resistance and virulence is promoted [47,62]. The pressure for selection of resistant microbes is probably higher in healthcare settings due to the turnover of many sick patients shedding along with administered antibiotics down the drains. Drains have been reported to be the source of several outbreaks. However, outbreaks are not often linked to the environment unless the outbreak isolate possesses an unusual multi-resistant profile [7]. The threat of mortality rate equaling the pre-antibiotic era is immediate and not for future. If no efficient antibiotics, current infections can’t be treated and sensitive interventions will not be possible to pursue. Therefore, preventive actions are urgent and must be taken to reduce infections causing us to focus on the fundamental need for a safe and maintained sanitation system.
